# Supplementary material for: Disruption of Endochondral Ossification and Extracellular Matrix Maturation in an Ex Vivo Rat Femur Organotypic Slice Model Due to Growth Plate Injury
Source: Cells. 2023 Jun 22;12(13):1687. doi: 10.3390/cells12131687 (PMC10341345; doi:10.3390/cells12131687)
Supplement: Supplementary file 1 [file cells-12-01687-s001.zip › cells-2408866-supplementary.pdf]

Supplementary

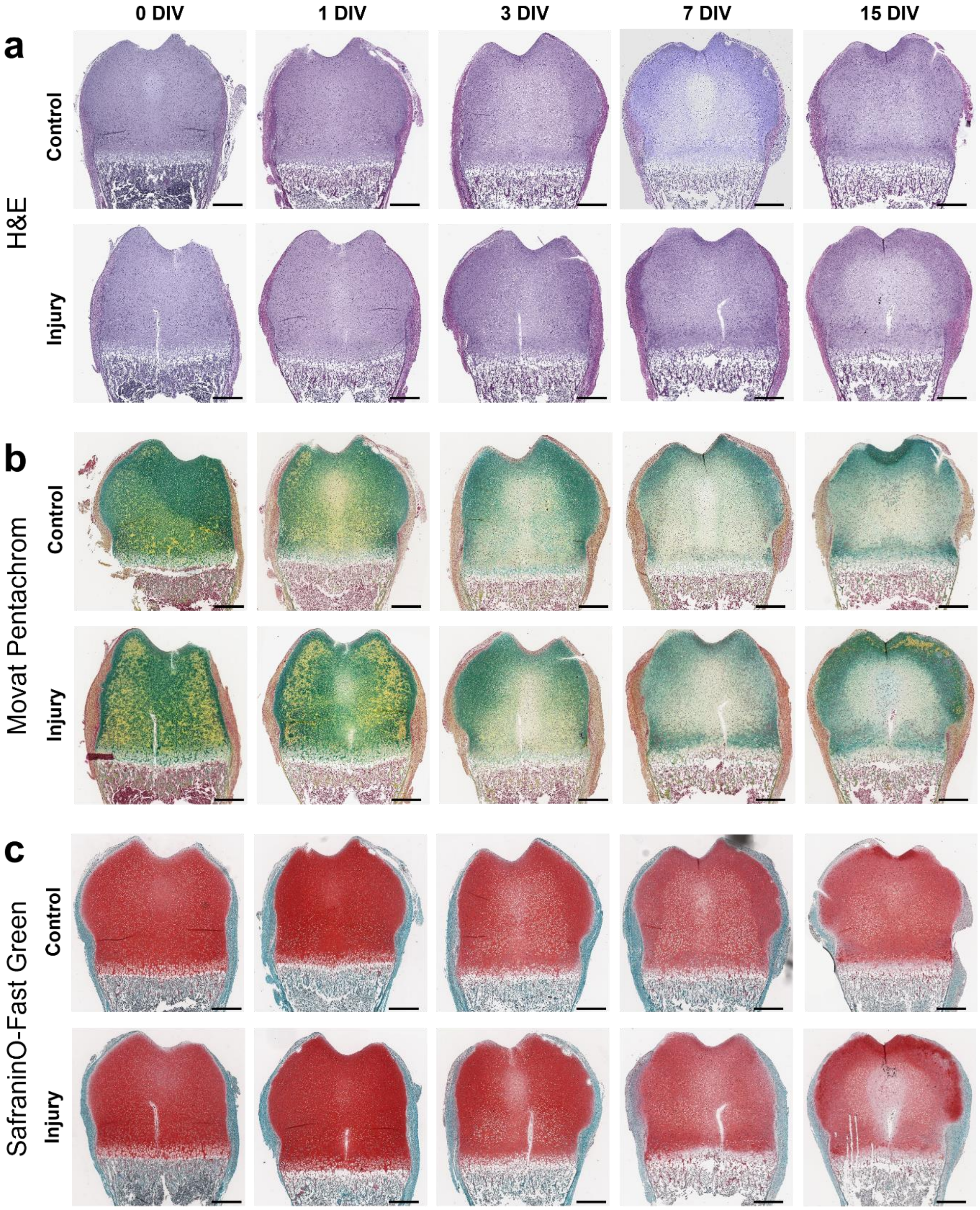

Supplementary Figure S1. Proximal rat femur of bone slices with and without growth plate injury were stained with different histological staining. Representative images of **(a)** Hematoxylin and eosin stain (H&E), **(b)** Movat Pentachrome and **(c)** SarfaninO-Fast Green stain of proximal rat femur of injured and non-injured control slices at 0, 1, 3, 7, and 15 days of *in vitro* culture (DIV). Scale bar = 500 µm.

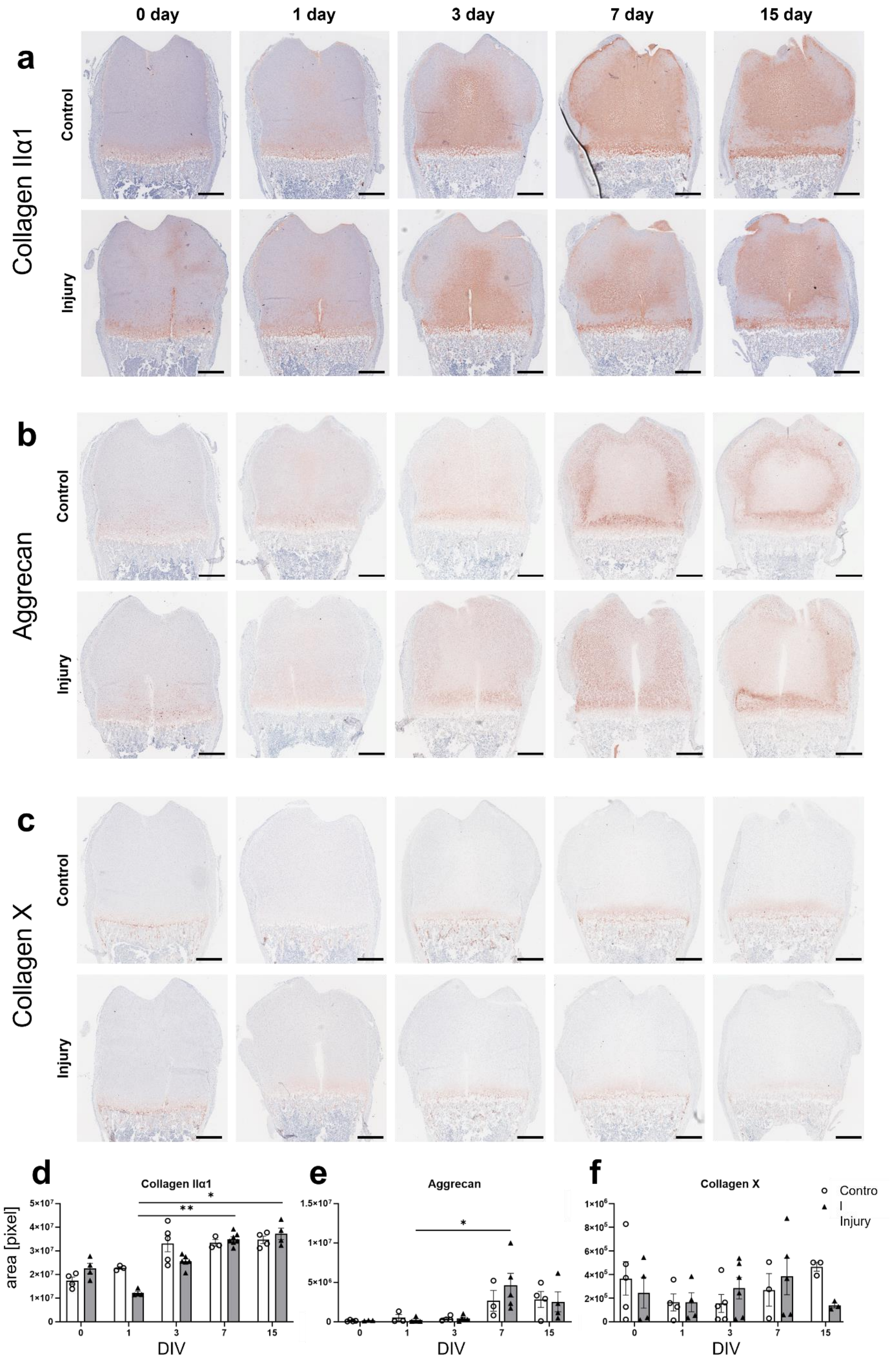
0 day
1 day
3 day
7 day
15 day
a
Collagen IIα1
Control
Injury
b
Aggrecan
Control
Injury
c
Collagen X
Control
Injury
d
Collagen IIα1
area [pixel]
DIV
e
Aggrecan
DIV
f
Collagen X
DIV
Contro
l
Injury

Supplementary Figure S2. Extracellular matrix (ECM) cartilage composition and chondrocyte maturation. (a-c) Maturation of chondrocytes are accompanied with the expression of type II collagen (Col2α1, committed chondrocytes), aggrecan (Acan, metabolic active chondrocyte) and type X collagen (ColX, hypertrophic chondrocytes); Scale bar = 500 µm. **(d-f)** Positive stained pixel within immunohistochemistry were quantified. Statistical significance of the observed differences was tested either with Kruskal-Wallis-Test (temporal changes) or with Mann-Whitney-U Test (injury vs. control). Data are summarized as the mean ± SEM and raw data were overlaid on histograms as triangles (injury) and circles (controls), n ≥ 3, *p ≤ 0.05, **p ≤ 0.01.

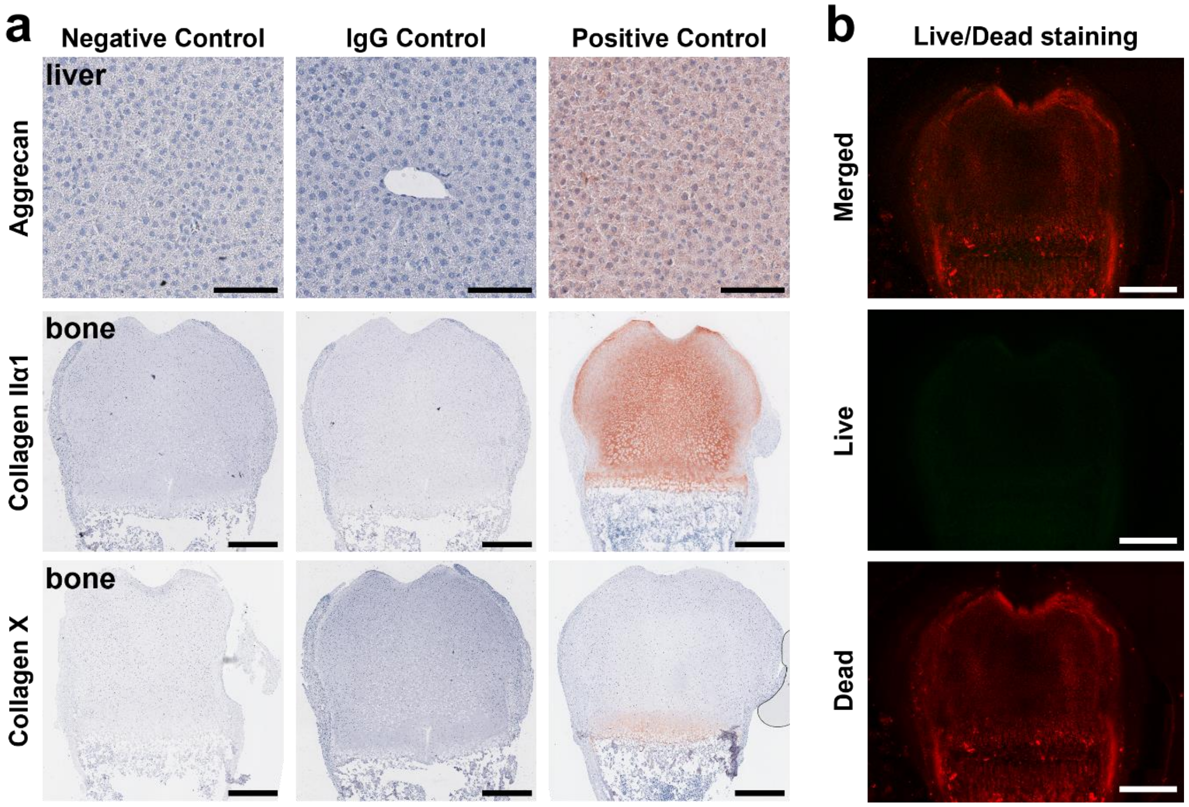

Supplementary Figure S3. Immunohistochemistry (IHC) and live/dead staining controls. (a) Immunohistochemistry controls with omission of primary antibody, IgG antibody in the same concentration as respective primary antibody and respective positive tissue control. Scale bar = 250 µm liver (aggrecan) and 300 µm bone slices (collagen IIα1 and collagen X). **(b)** Bone slice incubated in ethanol overnight served as dead positive controls. Scale bar = 500 µm.
